# Targeting Mast Cells Tryptase in Tumor Microenvironment: A Potential Antiangiogenetic Strategy

**DOI:** 10.1155/2014/154702

**Published:** 2014-09-11

**Authors:** Michele Ammendola, Christian Leporini, Ilaria Marech, Cosmo Damiano Gadaleta, Giovanni Scognamillo, Rosario Sacco, Giuseppe Sammarco, Giovambattista De Sarro, Emilio Russo, Girolamo Ranieri

**Affiliations:** ^1^Department of Medical and Surgery Sciences, Clinical Surgery Unit, University “Magna Graecia” Medical School, Viale Europa, Germaneto, 88100 Catanzaro, Italy; ^2^Department of Health Science, Clinical Pharmacology and Pharmacovigilance Unit and Pharmacovigilance's Centre Calabria Region, University of Catanzaro “Magna Graecia” Medical School, Viale Europa, Germaneto, 88100 Catanzaro, Italy; ^3^Diagnostic and Interventional Radiology Unit with Integrated Section of Translational Medical Oncology, Istituto Tumori “Giovanni Paolo II,” Viale Orazio Flacco 65, 70124 Bari, Italy; ^4^Radiotherapy Unit, Istituto Tumori “Giovanni Paolo II,” Viale Orazio Flacco 65, 70124 Bari, Italy

## Abstract

Angiogenesis is a complex process finely regulated by the balance between angiogenesis stimulators and inhibitors. As a result of proangiogenic factors overexpression, it plays a crucial role in cancer development. Although initially mast cells (MCs) role has been defined in hypersensitivity reactions and in immunity, it has been discovered that MCs have a crucial interplay on the regulatory function between inflammatory and tumor cells through the release of classical proangiogenic factors (e.g., vascular endothelial growth factor) and nonclassical proangiogenic mediators granule-associated (mainly tryptase). In fact, in several animal and human malignancies, MCs density is highly correlated with tumor angiogenesis. In particular, tryptase, an agonist of the proteinase-activated receptor-2 (PAR-2), represents one of the most powerful angiogenic mediators released by human MCs after c-Kit receptor activation. This protease, acting on PAR-2 by its proteolytic activity, has angiogenic activity stimulating both human vascular endothelial and tumor cell proliferation in paracrine manner, helping tumor cell invasion and metastasis. Based on literature data it is shown that tryptase may represent a promising target in cancer treatment due to its proangiogenic activity. Here we focused on molecular mechanisms of three tryptase inhibitors (gabexate mesylate, nafamostat mesylate, and tranilast) in order to consider their prospective role in cancer therapy.

## 1. Introduction

Angiogenesis is a complex process, mainly mediated by endothelial cells, consisting in the formation of new blood capillaries from existing vessels [1–4]. It is finely regulated by the balance between several angiogenesis stimulators, such as vascular endothelial growth factor (VEGF), fibroblast growth factor-2 (FGF-2), platelet derived growth factor (PDGF), angiopoietins, tryptase, and some angiogenesis inhibitors, including thrombospondin, angiostatin, and endostatin [5–11]. Angiogenesis, further than being involved in normal physiological processes, has been demonstrated to play a crucial role in cancer development inducing tumor growth, invasion, and metastasis [12, 13].

Mast cells (MCs) intervene in tissue angiogenesis through several classical proangiogenic factors such as VEGF, FGF-2, PDGF, interleukin-6 (IL-6), and nonclassical proangiogenic factors, such as tryptase and chymase, stored in their secretory granules [14–18]. In fact, MCs density is highly correlated with the extent of tumor angiogenesis both in benign tumors (e.g., in keloids) and in animal and human malignancies (systemic mastocytosis, head and neck, colorectal, lung, and cutaneous cancer) [19–24]. Tryptase and chymase stimulate angiogenesis and the response is similar to that obtained with VEGF [16]. This evidence confirms even more the angiogenic activity of these two proteases stored in MCs granules [16].

## 2. Role of Mast Cell Tryptase in Angiogenesis and Tumor Growth

MCs are tissue leukocytes originating from hematopoietic stem cells in bone marrow. Generally, these precursor cells circulate in blood as agranular cells; then, MCs migrate into different tissues completing their maturation into granulated cells under the influence of several microenvironmental growth factors. One of these crucial factors is the stem cell factor (SCF), the ligand of c-Kit receptor (c-KitR) secreted by fibroblasts and stromal and endothelial cells. SCF is critically involved in MCs activation [25, 26]. MCs can be naturally found in association with connective tissue structures (i.e., blood vessels, lymphatic vessels, and nerves) and in the proximity of skin and mucosa of the gastrointestinal, respiratory, and genitourinary tracts [27], which represent common portals of infections [26, 28]. Accordingly, for many years, MCs have been implicated in the pathogenesis of IgE-associated allergic reactions and certain protective responses to parasites, bacteria, viruses, and fungi [29–31]. However, increasing evidence suggests the involvement of these cells in several biological settings, such as inflammation, immunomodulation, angiogenesis, wound healing, tissue remodeling, and cancer [17, 32–41]. Specifically, the multiple functions of MCs depend on their capability to release panoply of biologically active products upon suitable immunological and nonimmunological stimulation [42]. These mediators are either preformed in their secretory granules (biogenic amines, neutral serine proteases) or synthesized* de novo* (metabolites of arachidonic acid, cytokines) [43, 44]. MCs granules represent key functional elements, whose content can be released by two distinct secretory mechanisms: exocytosis (*anaphylactic degranulation*) or* piecemeal degranulation* [25]. Interestingly, the latter process is the most frequent secretory mechanism observed in chronic inflammatory settings, such as cancer [31, 45].

A possible causal relationship between MCs, chronic inflammation, and cancer has long been suggested. Accordingly, as most tumors contain inflammatory cell infiltrates, often including abundant MCs, the question about the possible contribution of MCs to tumor development has progressively been emerging [31, 39]. MCs have been recognized as one of the earliest cell types to infiltrate many developing tumors, particularly malignant melanoma and breast and colorectal cancer (CRC) [8, 17, 21, 23, 40, 70, 71]. Ample evidence highlights that MCs accumulate predominantly around several types of tumors, at the boundary between malignant and healthy tissues [8, 17]. In particular, these cells are often strategically located in proximity of blood vessels within the tumor microenvironment, suggesting an early role of MCs in angiogenesis and tumor growth; in fact angiogenesis generates a new vascular supply that delivers oxygen and nutrients to the rapidly proliferating malignant tissue [25, 39, 72]. In agreement with this role, MCs are an abundant source of potent proangiogenic factors, which represent a major issue linking these cells to cancer [26, 73]. In many experimental tumor settings, MCs promote angiogenesis by releasing preformed mediators or by activating proteolytic release of extracellular matrix-bound angiogenic molecules [25, 32, 72].* In vitro* studies have demonstrated that MC granular components can induce vascularization [25]. Indeed, the addition of either human recombinant tryptase or chymase is able to stimulate neovascularization in the chick embryo chorioallantoic membrane assay (CAM) [32, 72]. Based on these results, treatment with cromolyn, an inhibitor of MCs degranulation, has been shown to restrain expansion and survival of pancreatic cancer and endothelial cells [15].

**Figure 1:**
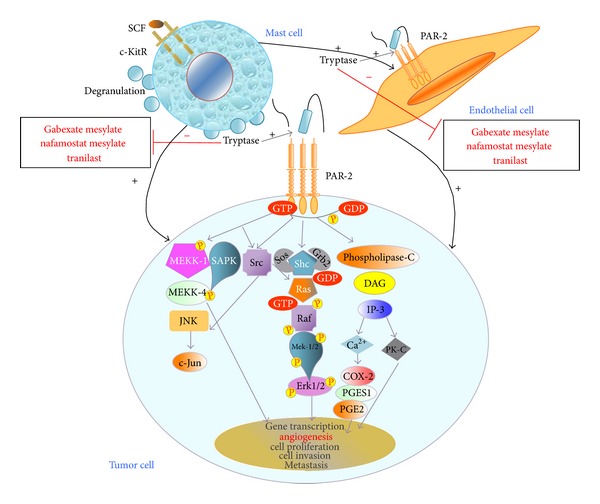
Tryptase, released after MCs activation of c-KitR/SCF-mediated, acting on PAR-2 by its proteolytic activity, has angiogenic activity stimulating both human vascular endothelial and tumor cell proliferation in paracrine manner, helping tumor cell invasion and metastasis. In cancer treatment, tryptase may represent a promising target by tryptase inhibitors (gabexate mesylate, nafamostat mesylate, tranilast) due to their potential antiangiogenic activity. c-KitR, c-Kit receptor; PAR-2, proteinase-activated receptor-2; VEGFR, vascular endothelial growth factor receptor; SCF, stem cell factor, VEGF, vascular endothelial growth factor; NHERF-1, Na+/H+ exchanger regulatory factor-1; MEKK-1, mitogen-activated protein kinase/extracellular signal-related kinase-1; MEKK-4, mitogen-activated protein kinase/extracellular signal-related kinase-4; JNK, c-Jun N-terminal kinase; c-Jun, Jun protooncogene; SAPK, mitogen-activated protein kinase-9; GEF, rho/rac guanine nucleotide exchange factor; Rho, rhodopsin transcription termination factor; SOS, SOn of sevenless protein; Grb2, growth factor receptor-bound protein 2; Shc, Shc transforming protein kinase; Ras, Ras protein kinase; Raf, Raf protein kinase; mitogen-activated protein kinase/extracellular signal-related kinase-1/2; Erk, Elk-related tyrosine kinase; DAG, Diacylglycerol; IP-3, inositol triphosphate; PK-C, protein kinase-C; COX-2, cyclooxygenase-2; PGE2, prostaglandin E2; PGES-1, prostaglandin E synthase-1; PK-A, protein kinase-A.

Tryptase and chymase are preformed active serine proteases and are stored in large amounts in MCs secretory granules [74], whose angiogenic role has been established [16, 75]. In particular, tryptase represents one of the most powerful angiogenic mediators released by human MCs upon c-KitR activation, and it may be angiogenic via several mechanisms [24]. This protease directly stimulates human vascular endothelial cell proliferation acting on protease-activated receptor-2 (PAR-2) by its proteolytic activity [24, 75, 76], leading to direct angiogenic effect (Figure 1). This particular proliferative pathway has been showed by Yoshii et al. [50] who have demonstrated that tryptase induces PAR-2-mediated proliferative effects on a human colon carcinoma cell line (DLD-1 cells) in a mitogen-activated protein kinase (MAP) kinase- and cyclooxygenase- (COX-) dependent manner. PAR-2 activation also leads to the release of IL-6 and granulocyte-macrophage colony stimulating factor (GM-CSF), which, in turn, act as angiogenic factors [77]. The important role of tryptase in neovascularization is also shown by its ability to degrade connective tissue matrix in order to provide rooms for neovascular growth. Tryptase may also contribute indirectly to tissue neovascularization by activating latent matrix-metalloproteinases (MMPs) and plasminogen activator, which, in turn, degrade extracellular matrix (ECM) with consequent release of ECM-bound angiogenic factors, such as VEGF and FGF-2 [25, 26, 52]. The disruption of local ECM leads also to release of SCF. Interestingly, tumor-derived SCF has been recently implicated both in MCs recruitment into the tumor environment as well as in increased MCs release and production of VEGF and FGF-2 [78, 79].

With reference to the above-described mechanisms that link tryptase to tumor angiogenesis and cancer progression, several studies have reported a linear correlation between mast cells density positive to tryptase (MCDPT) and angiogenesis in solid tumors, such as human malignant melanoma [80, 81], endometrial carcinoma [41], breast cancer [8, 82], uterine leiomyomas [83], gastric cancer [23, 24, 40], and CRC [21, 84]. Regarding hematological tumors, angiogenesis has been shown to increase with the MCDPT in B cell non-Hodgkin's lymphomas [85] as well as in the bone marrow of patients with multiple myeloma, monoclonal gammopathies of undetermined significance [86], myelodysplastic syndrome [87], and B-cell chronic lymphocytic leukemia [85]. In the majority of studies, MCDPT correlates with angiogenesis, tumor aggressiveness, and poor prognosis [25], even if some human studies have demonstrated a correlation between high mast cells density (MCD) and improved overall survival [88–91], suggesting that MCs effects on tumor fate may depend on some* bias* related to cancer (e.g., type of surgical treatment with relative lymph node collection, histology, stage tumor, small sample size) and different methods of MCs evaluation (e.g., histochemistry with toluidine blue, Giemsa stain, primary antibody antitryptase or antichymase for immunohistochemistry, standardization of MCs count with reference to magnification, MCs location, and microscopic field of evaluation).

Overall, despite conflicting reports on the role of MCD- and MCDPT-mediated angiogenesis in tumor development, literature data indicate that tryptase may represent a promising target in adjuvant cancer treatment [25, 26], leading to considering the therapeutic use of drugs which specifically inhibit its angiogenic activity. Therefore, tryptase inhibitors, such as gabexate mesylate and nafamostat mesylate [92–94], might be evaluated in clinical trials as new antiangiogenic agents in combination with chemotherapy in the treatment of cancer.

## 3. Potential Role of Mast Cells Tryptase Inhibitors in Cancer

In the light of the aforementioned complex relationship between MCs tryptase and angiogenesis in tumor development, we have described the possible molecular mechanisms of three drugs targeting tryptase functions, such as gabexate mesylate, nafamostat mesylate, and tranilast, in order to discuss their prospective role in cancer therapy.

### 3.1. Gabexate Mesylate

Gabexate mesylate (GM) is a synthetic inhibitor of trypsin-like serine proteases [95–97] that shows an antiproteinase activity on various kinds of plasma proteases, such as thrombin, plasmin, trypsin, kallikrein, C1 esterase in the complement system, and factor Xa in the coagulation cascade. Accordingly, GM has been therapeutically used for disseminated intravascular coagulation (DIC) and acute pancreatitis [95] in Japan, Italy, Korea, and Taiwan. In addition, several recent studies have reported that this protease inhibitor exerts a significant antitumorigenic effect, both* in vitro* and* in vivo* [46, 47, 94].

Proteolytic degradation of ECM components is a crucial step for tumor cell invasion and metastasis. Among several classes of degrading ECM proteinases, MMPs (MMP-2 and MMP-9) and urokinase-type plasminogen activator (uPA) have been closely associated with the metastatic phenotype of cancer cells [98–102]. These enzymes are also implicated in tumor angiogenesis [103]. Therefore, inhibitors of MMPs and uPA are able to inhibit invasion and metastasis [104, 105] by reducing angiogenesis* in vitro* and* in vivo* [106–109]. Furthermore, serine plasma proteases, such as thrombin and plasmin, are closely associated with activation pathways of certain MMPs (MMP-2, MMP-3, and MMP-9) [110, 111], indicating that multispecific protease inhibitors could be useful tools for an antimetastatic and antiangiogenic strategy. Based on these findings, GM has been shown to inhibit proliferation, invasion, and metastasis of human colon cancer cell lines through the inhibition of both MMPs and uPA-plasmin system, consequentially limiting angiogenesis [46]. Although the inhibition of the uPA system may be involved in downregulation of MMP activity, the results of this study have suggested that GM has a direct inhibitory effect on MMPs, whose related-mechanism is unknown [46].

Interestingly, the inhibition of MMPs by GM and, in general, its anti-invasive, antimetastatic, and antiangiogenic properties could also be explained through its potent and selective inhibition of human tryptase [92]. Indeed, as above described, in the early stages of tumor development several tumor-derived factors (i.e., SCF, adrenomedullin) recruit and activate MCs in tumor microenvironment, leading to the release of tryptase [25, 26], which, in turn, can indirectly stimulate tumor angiogenesis by activating latent MMPs and uPA [23, 24, 75]. Accordingly, Yoshii et al. [50] demonstrated the specific localization of MCDPT in the invasive front of tumor tissues by examining 30 cases of human colon adenocarcinoma. A previous study [49] has found the proliferation of DLD-1 colon cancer cells expressing PAR-2 in response to PAR-2 activating peptide (AP). Moreover, tryptase also enhanced DLD-1 cell proliferation by means of a specific stimulation of PAR-2 via MAPK- and COX-dependent manners. Furthermore, these proliferative effects were concentration-dependently inhibited by nafamostat mesylate, a very potent inhibitor of human tryptase [93, 112], suggesting that PAR-2 activation was dependent on tryptase proteolytic activity. In the same study, PAR-2 density in tumor tissues was higher than that in the normal tissues, as revealed by the immunohistochemical analysis. This suggests that tryptase released by MCs surrounding tumor tissues may induce the PAR-2-mediated proliferation of colon cancer cells in a paracrine way [49]. Similarly, tryptase has been reported to stimulate angiogenesis directly [75] via PAR-2 activation on vascular endothelial cells [24, 76]. Moreover, increasing evidences support that MCs tryptase is involved in angiogenesis through the direct degradation of connective tissue matrix [25, 26, 75], with consequent release of matrix-associated angiogenic substances, such as VEGF or FGF-2 [40, 72, 113–116]. These findings as a whole suggest that MCs tryptase may sustain colon cancer cell growth in two ways: direct proliferative effect via PAR-2 stimulation and indirect support through angiogenesis stimulation. Thus, tryptase may be considered a novel target of colon cancer therapy. Taken together, all the reported evidences suggest that the inhibition of colon cancer growth, invasion, and metastasis by GM may be also due to its selective inhibition of MC tryptase. Therefore, GM could be potentially useful for antimetastatic and antiangiogenic treatment of colon cancers. Noteworthy, this assumption is corroborated by a recent study by Brandi et al. [47] aimed to investigate the antitumor efficacy of GM, alone, and in combination with the antiepidermal growth factor receptor (EGFR) monoclonal antibody cetuximab, in a group of human CRC cell lines with a different expression pattern of wild-type/mutated V-Ki-ras2 Kirsten rat sarcoma viral oncogene homolog (K-RAS), protooncogene B-Raf murine sarcoma viral oncogene homolog B1 (BRAF), and phosphatidylinositol-4,5-bisphosphate 3-kinase, catalytic subunit alpha oncogene (PIK3CA). Besides confirming the lack of response to cetuximab in CRC cells bearing such mutations [117–119], results demonstrated that GM significantly inhibited the growth, invasiveness, and tumor-induced angiogenesis in all CRC cells tested in this study [47]. In particular, the antiangiogenic effect of GM in combination with the anti-EGFR antibody was found to be not superior than that observed with GM as single agent, suggesting that the inhibition of tumor angiogenesis may be largely related to GM mechanism of action, most notably the inhibition of MCs tryptase. Therefore, also considering its good toxicological profile, these findings indicate that GM could represent a valuable therapeutic option for patients with EGFR-expressing metastatic CRC (mCRC), particularly for those ones bearing KRAS, BRAF, and PIK3CA mutations, either as monotherapy or in combination with standard chemotherapy [47].

The antimetastatic and antiangiogenic mechanisms of GM have also been investigated in pancreatic cancer cell lines. As in colon cancer, MMPs and uPA play a crucial role also in the progression of pancreatic cancer [48]. In addition, a previous study by Uchima et al. [48] reported the involvement of tumor-associated trypsinogen (TAT) and pancreatic acinar trypsinogen (PAT) in pancreatic cancer invasion and metastasis. Both these serine proteases can be activated by uPA, which is produced by pancreatic cancer. Following, they are able to degrade ECM components and can also directly activate TAT, PAT, pro-MMPs, and pro-uPA, leading to further ECM breakdown. The resulting* vicious cycle* would activate latent ECM-degrading proteases, thereby promoting tumor cell invasion and metastasis. In particular, PAT and TAT had been shown to continuously stimulate pancreatic cancer cell proliferation by activating PAR-2 [120]. Furthermore, several investigations reported that transforming growth factor-beta 1 (TGF-*β*1), produced in the tumor microenvironment, could be a strong mediator of pancreatic cancer cell invasion, metastasis, and angiogenesis by upregulating VEGF, MMP-2, and uPA secretion [121–123]. High uPA levels, in turn, could activate latent TGF-beta1, resulting in a positive feedback loop on tumor progression [123]. Starting from these data, Uchima et al. [94] suggested that GM inhibited the invasiveness, proliferation, and potential liver metastatic of pancreatic cancer cell lines by downregulating TAT and uPA activities, reducing PAR-2 activation, and inhibiting the production of TGF-*β*1 and VEGF. Moreover, in pancreatic cancer the inhibitory effects of GM may be, in part, associated with tryptase inhibition. In fact, similarly to TAT, MCs tryptase may be responsible for PAR-2-mediated pancreatic cell proliferation, since tryptase is a natural agonist of this receptor [124]. Moreover, tryptase-mediated activation of latent MMPs and uPA [25, 26, 75] may induce further TAT, MMPs, and uPA activation and ECM degradation, thus triggering an ECM-protease network responsible for tumor cell invasion and metastasis [48, 94]. In this context, tryptase inhibition by GM may downregulate TAT and uPA enzymatic activities. The resulting downregulation of uPA levels may decrease the activation of latent TGF-*β*1, thereby impairing the abovementioned* cycle vicious* of uPA and TGF-*β*1 and downregulating VEGF production. On the other hand, tryptase inhibition may also directly suppress the production of TGF-*β*1 and VEGF involved in tumor growth and angiogenesis. In agreement with this proposed mechanism, tryptase has been reported to increase the production of TGF-*β*1 in other pathophysiological settings [125, 126]. The findings about the GM mechanism of action in pancreatic cancer cells, together with our considerations, indicate that this protease inhibitor could be a useful therapeutic option for antimetastatic and antiangiogenic treatment of pancreatic cancer.

The above studies are summarized in Table 1.

### 3.2. Nafamostat Mesylate

Similarly to GM, nafamostat mesylate (NM) is able to inhibit a variety of trypsin-like serine proteases and some proteases implicated in the coagulation cascade [127, 128]. Interestingly, Mori et al. [93] have demonstrated that NM inhibits human tryptase with potency 1000 times higher than that of GM, concluding that NM is an extremely potent and selective inhibitor when employed at relatively low concentration. They have also suggested that such inhibitory action on tryptase activity can account for some therapeutic effects of NM in specific clinical conditions. Indeed, human tryptase may be involved in the pathogenesis of several MCs-mediated allergic and inflammatory diseases, such as rhinitis and asthma. It is also implicated in specific gastrointestinal, dermatological, and cardiovascular disorders [129–131]. Therefore, NM has been widely used for the treatment of acute pancreatitis and DIC in Japan [132, 133].

The antitumor potential of NM is suggested by Yoshii et al.'s study previously described [50]. In fact, the* in vitro* analysis showed that NM concentration-dependently inhibited the tryptase-induced enhancement of proliferation of DLD-1 cells, thus suggesting that tryptase inhibition may mediate the anticancer effect of NM. It has also been reported that NM inhibits liver metastases of colon cancer cells in mice [134]. Moreover, previous studies showed that NM inhibited the proliferation and invasion of pancreatic cancer cells by antagonizing TAT-induced activation of PAR-2* in vitro*, in the same fashion of GM [51, 52]. Indeed, several studies have recently revealed that NM exerts antiproliferative, antiangiogenic, and antimetastatic effects also in pancreatic cancer, proposing the use of this serine protease inhibitor in combination with standard chemotherapy regimens for pancreatic cancer management [53–56]. In particular, the blockade of nuclear factor kappa-B (NF-*κ*B) activation has been reported to underlie antitumor effects of NM [55]. In this regard, Karin and Lin have demonstrated that NF-*κ*B plays an important role in the modulation of inflammatory responses, cell proliferation, apoptosis, and oncogenesis including invasion and angiogenesis [55]. Typically, inactive NF-*κ*B is sequestered in the cytoplasm by nuclear factor of kappa light polypeptide gene enhancer in B-cells inhibitor alpha (IkB*α*); however, a specific activation signaling leads to IkB*α* phosphorylation and consequent release of NF-*κ*B protein, which translocates into the nucleus, where it induces the transcription of target genes [135]. Most notably, constitutive activation of NF-*κ*B has been identified in a variety of tumors including pancreatic cancer [135] and it is known to contribute to the aggressive phenotype [136] and chemoresistance [137]. The resulting overexpression of downstream target genes of NF-*κ*B, such as intercellular adhesion molecule-1 (ICAM-1) [138], IL-8 [136, 139], VEGF [136, 139, 140], MMP-9 [140], and uPA [141], promotes cell adhesion, angiogenesis, invasion, and metastasis. Interestingly, some cancer chemotherapy drugs, such as oxaliplatin and gemcitabine, have been shown to activate NF-*κ*B by themselves, thereby reducing their antitumor efficacy [142–144]. Based on these findings, a NF-*κ*B inhibitor like NM may be able to suppress proliferation, angiogenesis, and metastasis both in pancreatic cancer and in other malignancies, opening an avenue for novel therapeutic approaches. In the study by Fujiwara et al. [55], NM has been shown to downregulate activities of phosphorylated IkB*α*, NF-*κ*B, and its target genes, resulting in inhibition of cell adhesion, invasion, and increase of a particular programmed cell death (*anoikis*) in human pancreatic tumor cell lines.* In vivo*, intraperitoneal administration of pancreatic cancer cells, pretreated with NM, in nude mice revealed reduced peritoneal metastasis and neovascularization and increased survival compared with controls. This suggests that NM may potentially reduce the incidence of postoperative recurrences due to peritoneal dissemination in pancreatic cancer patients [145]. In accordance with these findings, the authors have already reported the ability of NM to inhibit NF-*κ*B activation and induce caspase-8-mediated apoptosis when this serine protease inhibitor was used as monotherapy or with gemcitabine,* in vitro* and* in vivo* [53, 146, 147]. Most notably, they reported a better clinical outcome of combination therapy of gemcitabine or paclitaxel with NM in comparison with gemcitabine or paclitaxel alone in pancreatic cancer-bearing mice through the inhibition of chemotherapeutic drug-induced NF-*κ*B activation [53, 55]. It was also demonstrated the clinical usefulness of intra-arterial NM administration combined with gemcitabine in patients with unresectable pancreatic cancer [54, 148]. Accordingly, Gocho and colleagues [56] have recently proven that NM enhances the antitumor effect of oxaliplatin by inhibiting oxaliplatin-induced NF-*κ*B activation. This leads to downregulation of the cellular inhibitor of apoptosis proteins, c-IAP1 and c-IAP2, resulting in cleavage of poly ADP-ribose polymerase (PARP) and caspase-8-mediated apoptosis* in vitro* and* in vivo*: the inhibition of NF-*κ*B activity results in chemosensitization of pancreatic cancer. Therefore, combination chemotherapy with NM and oxaliplatin exerts a synergistic cytotoxic effect in pancreatic cancer both* in vitro* and* in vivo*.

Taking into account the above-illustrated pathophysiological pathways, we propose that the potent inhibition of MCs tryptase may also be involved in the antitumor activities of NM. Firstly, this hypothesis is supported by the ability of tryptase to stimulate cell proliferation and invasion of cancer cells* in vitro* through the activation of PAR-2. We herein report the evidence of these tryptase-mediated proliferative effects only in colon cancer cells [50]; however, tryptase, being a natural agonist of PAR-2 [124], may be potentially able to activate this receptor class expressed also in the gastrointestinal tract, pancreas, liver, kidney, and sensory neurons [149–151], triggering a proliferative response. Moreover, the above mentioned antiproliferative effect of NM in pancreatic cancer cells by blocking TAT-induced PAR-2 stimulation [51, 52] may be indirectly related to tryptase inhibition. In fact, we have previously reported that tryptase can activate the uPA system [25, 26, 75], which, in turn, activates TAT leading to stimulation of PAR-2 on the surface of pancreatic cancer cells [94]. On the other hand, the inhibition of tryptase-mediated activation of PAR-2 on vascular endothelial cells could contribute to antiangiogenic effects of NM.

MCs tryptase may contribute to cancer pathways triggered by the constitutive activation of NF-*κ*B. In particular, tryptase may upregulate the levels of several target genes overexpressed owing to the pathological NF-*κ*B activation, such as VEGF, IL-8, MMP-9, and uPA, thereby contributing to promote angiogenesis, invasion, and metastasis in a variety of tumors. Interestingly, several studies have reported that PAR-2 is able to mediate some important tryptase-induced inflammatory processes, such as microglia activation and skin inflammation [152, 153]. In particular, it has been shown that MC tryptase, via PAR-2, may induce the upregulation/release of proinflammatory cytokines (i.e., IL-6, IL-8, TNF-*α*) and activate important inflammatory signaling cascades such as NF-*κ*B pathway in human dermal microvascular endothelial cells and microglia: MAPK signaling pathways are involved in NF-*κ*B activation and consequent production/release of proinflammatory cytokines by tryptase [152, 153]. Furthermore, according to Ma et al. [154] tryptase could phosphorylate protein-kinase B (PKB, also known as AKT) through PAR-2, activate phosphoinositol-3-kinase (PI3K)/PKB pathway, and upregulate the expression of NF-*κ*B in inflammatory settings. Most notably, PKB/AKT is involved in cellular survival pathways by inhibiting apoptotic processes [155]; hence, it has been implicated as a major factor in many types of cancers [156].

In the light of these last findings, MCs tryptase may probably contribute to the aggressive behavior and chemoresistance of pancreatic cancer cells, by activating NF-*κ*B. Therefore, the inhibitory effect of NM on NF-*κ*B activities may also indirectly depend on the selective tryptase inhibition. On the other hand, tryptase inhibition could also justify the apoptotic effect of NM through the downregulation of PI3K/protein kinase B (PKB) signaling pathway. As a whole, the above detailed findings and mechanisms suggest a potential usefulness of NM in preoperative management of pancreatic cancer patients, because its use may reduce postoperative recurrences and improve survival by inhibition of metastasis induced by surgical resection [157]. Moreover, taking into account the improved outcomes and relatively low toxicity of preclinical and clinical studies of the combination therapy with traditional chemotherapeutic agents and NM, these combination chemotherapy regimens could represent a novel promising strategy for pancreatic cancer treatment.

The above studies are summarized in Table 2.

### 3.3. Tranilast

Among pharmacological agents that affect several inflammatory and allergic pathways mediated by MCs tryptase, also tranilast (TN) has progressively attracted considerable attention because of its antitumor potential. Since 1982, this drug has been approved in Japan and Korea for the systemic and topical treatment of bronchial asthma, atopic dermatitis, and allergic conjunctivitis, with indications for keloids and hypertrophic scar added in 1993 [158]. Follow-up studies have revealed that clinical effectiveness of TN in such applications depends on inhibition of the release of biologically active mediators from MCs [158, 159]. Moreover, tranilast was reported to inhibit the VEGF-induced angiogenesis both* in vitro* and* in vivo*, and most notably, these antiangiogenic activities have been shown to be concomitant with inhibitory effects on MCs degranulation [160].

TN was also reported to inhibit the release of TGF-beta, IL-1beta, prostaglandin (PG) E_2_, and IL-2 from human monocytes and macrophages [161, 162]. In the late 1980s, Isaji et al. [160] discovered the antiproliferative properties of TN. In particular, it was found that this agent inhibited fibroblast proliferation* in vitro*, resulting in suppression of proliferative inflammation* in vivo*. Subsequent studies confirmed the ability of TN in inhibiting tumor cell growth and proliferation in various models of cancer [59, 163, 164]. Overall, data from* in vitro* and* in vivo* models for proliferative disorders, clinical studies, and case reports have corroborated the antiproliferative and antitumor potential of TN [165], providing important insights into its mechanisms of action. Two studies, addressing antiproliferative activity of TN in several breast cancer cell lines, revealed that TN inhibits cell proliferation, by arresting cell cycle progression, and downregulates TGF-*β* signaling pathway [57, 58]. Moreover, Chakrabarti et al. [57] demonstrated that TN is able to inhibit MAPK signaling pathway.

TN was also reported to suppress the proliferation of cultured human leiomyoma cells by inhibiting cell cycle modulators, such as cyclin-dependent kinase 2 (CDK-2) [164].

As concerns pancreatic cancer, Hiroi et al. [59] reported that TN significantly inhibited proliferation of PGHAM-1, a hamster pancreatic cancer cell line. Moreover, TN was able to inhibit tumor angiogenesis in response to VEGF. Interestingly, in another study by Mitsuno et al. [60] TN was found to enhance chemotherapeutic effect of gemcitabine, as above reported for NM [53, 54]. However, unlike NM-induced effect, this chemosensitization was associated with the downregulation of ribonucleotide reductase M1 (RRM1) [53, 54].

Further experiments revealed that TN treatment inhibited prostate cancer cell proliferation* in vitro* by promoting apoptosis. In addition, it was reported the ability of TN to downregulate TGF-beta production from bone stromal cells and other different cell types, thereby suppressing TGF-*β*-stimulated osteoclast differentiation which underlies, in part, osteoblastic bone metastasis [61, 166]. Noguchi et al. [62] demonstrated that three weeks of TN treatment significantly reduced the tumor growth and metastasis, when administered daily by intraperitoneal injection (4 mg/animal), in a mouse model of oral squamous cell carcinoma. TN has also been reported to exert antitumor effects in gastric cancer [63] and malignant glioma [64] through different mechanisms. Izumi et al. [61] have reported that the treatment with oral TN (300 mg/day) promoted a reduction of prostate-specific antigen (PSA) levels in 4 out of 16 patients with advanced castration-resistant prostate cancer (CRPC). Accordingly, in the subsequent follow-up pilot study, oral treatment with TN (300 mg/day) for a median period of five months documented a continuous PSA inhibition in 3 out of 21 patients with advanced CRPC. Overall survival rates at 12 and 24 months were 74.5% and 61.5%, respectively [167]. As a whole, these results suggest that TN could be used to improve the prognosis of patients with advanced CRPC. However, the two clinical investigations had some limitations: (1) open-label studies with one arm; (2) short follow-up period; (3) small sample size; (4) all patients were Japanese. Therefore, the reported findings need further confirmation. Finally, several case studies have reported that transdermal application of TN was able to relieve both itching and pain associated with hypertrophic, keloid scars [168].

Several important pathways have been recognized as potential targets of TN antitumor activity. In particular, the TN inhibitory effects on cell proliferation depend mainly on its ability to interfere with TGF-*β* signaling and also reduce TGF-*β* secretion [57, 61, 63, 64]. Also, TN-mediated inhibition of cell proliferation has been markedly associated with blockade of cell cycle progression and consequent cell arrest in the *G*_0_/*G*_1_ transition [58, 59, 164, 169]. Probably, TN can induce cell cycle arrest also through the inhibition of calcium influx, which is crucial for *G*_1_/*S* transition, as demonstrated by Nie et al. [65]. After TN treatment, the induction of apoptosis has been reported in several breast and prostate cancer cell lines [61, 66]. In particular, Subramaniam et al. [58] showed that TN induced p53 upregulation, enhanced RAC-alpha serine/threonine-protein kinase (AKT1) phosphorylation, and reduced phosphorylation of extracellular regulated kinase 2 (ERK2). Another work by Subramaniam et al. [66] detected an increased level of a PARP-cleavage product in human cancer cell lines treated with TN.

TN also acts as a nontoxic agonist of the aryl hydrocarbon receptor (ARH) [58, 170], whose function is involved in anticancer effects [67, 68]: ARH presence in the cell is critical for TN-mediated cell cycle arrest. Interestingly, the AHR also antagonizes TGF-*β* activity [171] and exerts ligand-dependent inhibitory effects on NF-*κ*B signaling [172]. These AHR-mediated activities may contribute to the antiproliferative, antiangiogenic, and antimetastatic effects of TN.

The ability of TN to inhibit MAPK signaling pathway could also explain its antimetastatic potential, because this pathway is known to be implicated during the epithelial to mesenchymal transition (EMT), which is important for tumor cell invasion [57]. Moreover, the downregulation of certain MMPs, such as MMP-9, contributes to TN-mediated inhibition of tumor cell invasion during metastasis: such reduction of MMP-9 levels has been also linked to inhibition of TGF-*β* signaling [58].

In addition to the above detailed potential targets, the antitumor action of TN relies on the blocking of the release of chemical mediators from MCs [57, 61, 64, 159], which is also the mechanism responsible for its antiallergic and anti-inflammatory efficacy [158]. In agreement with this correlation, Yamamoto et al. [158] have recently documented that TN downregulated neurofibroma cell (NF1 cells) proliferation through not only suppression of cell-growth promoting pathways but also the inhibition of biologically active mediators by MCs. Interestingly, this study supports the involvement of tryptase in the antitumor activities of TN. Indeed, following its addition to NF1 cells cocultured with MCs, this agent was reported to significantly inhibit NF1 cell proliferation and lower the levels of TGF-*β*, SCF, and tryptase. These findings suggest that TN inhibits tumor proliferation also through the downregulation of MC tryptase, whose PAR-2-mediated proliferative and angiogenic effects have been previously described [50, 76]. Furthermore, tryptase has been reported to activate PI3K/PKB pathway via PAR-2 cleavage/activation and subsequently upregulate NF-*κ*B expression [154], promoting tumor cell survival and chemoresistance [56, 155, 156]. Thus, the inhibition of tryptase release may represent a further molecular mechanism involved in the induction of apoptosis and cell cycle arrest upon TN treatment. Because tryptase-mediated PAR-2 activation triggers the MAPK signaling pathway, which is involved in the EMT process [57], tryptase inhibition by TN may also mediate its anti-invasion and antimetastatic properties.

The inhibitory effect on tryptase release could contribute to the ability of TN treatment to target TGF-beta-regulated signaling cascade and reduce TGF-*β* production. As above described, indeed, in the tumor microenvironment, tryptase may upregulate uPA levels, [25, 26, 75] thereby activating latent TGF-*β* which, in turn, upregulates the production of uPA, MMP-2, and VEGF. This* vicious cycle* has been implicated in angiogenesis, tumor cell invasion, and metastasis [94]. By the way, tryptase can also participate to the neovascular growth by activating latent MMPs, which, in turn, promote tumor invasiveness and release of angiogenic factors (VEGF or FGF-2) from their matrix-bound state [25, 26, 75]. Therefore, also taking into account the previously reported study by Isaji et al. [69], the downregulation of tryptase release may probably contribute to the TN-induced inhibition of tumor angiogenesis in response to VEGF, as observed in experimental pancreatic cancer [59].

In the light of the exposed considerations, we suggest that the inhibition of tryptase functions may underlie the anti-invasion, antimetastatic, and antiangiogenic effects of TN treatment. As concerns safety, TN shows relatively low toxicity in [61, 69, 173], making it a promising candidate for further clinical investigations. Based on the encouraging* in vitro* and* in vivo* research data, TN seems to be a safe and effective agent for the treatment of several proliferative and angiogenic diseases.

The above studies are summarized in Table 3.

## 4. Concluding Remarks

Several literature data support a potential implication of MCs tryptase in three pivotal processes involved in cancer development and metastasization: cell growth, tumor-induced angiogenesis, and invasion [174, 175]. Therefore, this serine protease may be considered a novel promising target for the adjuvant treatment of tumors through the selective inhibition of angiogenesis, proliferation, and tissue remodelling. In agreement with these considerations, compounds targeting tryptase functions, although designed as antiallergic drugs, could exert a useful antitumor activity as well. In this regard, it is of interest to underline that many new anticancer drugs used in clinical field, such as sorafenib [18], sunitinib [176], pazopanib [177] axitinib [178], and masitinib [179] are all targeted against c-KitR, whose activation leads to the release of tryptase by MCs [24].

In particular, we herein discuss the antitumor and antiangiogenic potential of three agents which are able to inhibit the functions of MCs tryptase: gabexate mesylate, nafamostat mesylate, and tranilast. Although no definitive experimental data are available to confirm the role that tryptase released from mast cells stimulate tumor angiogenesis, the above hypothesis is supported by a pilot study in the* in vivo* chorioallantoic membrane assay [16]. In this study an angiogenic activity of human recombinant tryptase comparable to the angiogenic activity induced by the VEGF has been demonstrated. Data from this study suggest that the inhibition of tryptase is intriguing hypothesis worthy to further investigation.

The new antiangiogenic approach here reviewed should be substantially strengthened by future awaited clinical studies having the aim to evaluate the truly efficacy of the tryptase inhibitors as a novel tumor antiangiogenic therapy.

## Figures and Tables

**Table 1:** All preclinical studies mentioned above that have considered gabexate mesylate.

Author, reference, year	Drug/s	Tumor target	Molecular mechanisms of action	Results
Yoon et al. [46] 2004	*gabexate mesylate *	several human colon cancer cell lines	(1) down-regulation of MMPs (2) inhibition of uPA-plasmin system	inhibition of angiogenesis, tumor cell growth, invasion, metastasis

Brandi et al. [47] 2012	(1)* gabexate mesylate* (2)* gabexate mesylate plus cetuximab *	several human colorectal cancer cell lines (wt/mut KRAS, BRAF, PIK3CA)	not analyzed	(1) inhibition of tumor cell growth, angiogenesis, invasion, metastasis (2) antitumoral efficacy of the combination therapy was not superior than gabexate mesylate alone

Uchima et al. [48] 2003	* gabexate mesylate *	several human pancreatic cancer cell lines	down-regulation of uPA, TAT, PAT, MMPs, TGF-*β*1, VEGF	inhibition of angiogenesis, cell growth, invasion, metastasis

MMPs, Metalloproteinases; uPA, urokinase-type plasminogen activator; wt, wild-type; mut, mutated; TAT, Tumor-associated trypsinogen; PAT, Pancreatic acinar trypsinogen; TGF-*β*1, Tumor growth factor-beta1; VEGF, Vascular endothelial growth factor.

**Table 2:** All studies mentioned above that have considered nafamostat mesylate.

Author, reference, year	Drug/s	Tumor target	Molecular mechanisms of action	Results
Jikuhara et al. [49] 2003	*nafamostat mesylate *	human colon cancer cell line (DLD-1)	(1) inhibition of PAR-2 stimulation via MAPK- and COX-dependent manner (2) inhibition of VEGF and FGF-2 levels	inhibition of tumor cell growth, angiogenesis, invasion, metastasis

Yoshii et al. [50] 2005	*nafamostat mesylate *	human colon cancer cell line (DLD-1)	(1) Inhibition of PAR-2 stimulation via MAPK- and COX-dependent manner (2) Inhibition of the release of IL-6 and GM-CSF	inhibition of angiogenesis, cell growth, invasion, metastasis

Tajima et al. [51] 2001	*nafamostat mesylate *	several human pancreatic cancer cell lines	antagonizing TAT-induced activation of PAR-2	inhibition of tumor cell growth and invasion

Ohta et al. [52] 2003	*nafamostat mesylate *	several human pancreatic cancer cell lines	antagonizing TAT-induced activation of PAR-2	inhibition of tumor cell growth and invasion

Uwagawa et al. [53] 2009	(1)* nafamostat mesylate* (2)* nafamostat mesylate plus gemcitabine *	human pancreatic cancer cell line (Panc-1)	Down-regulation of NF-*κ*B with reduction of ICAM-1, IL-8, VEGF, MMP-9, uPA, RRM1	(1) inhibition of tumor cell adhesion and growth, angiogenesis, invasion metastasis (2) increase of apoptosis (3) increase of body weight loss of mice

Uwagawa et al. [54] 2009	*nafamostat mesylate plus intra-arterial gemcitabine *	unresectable locally advanced or metastatic pancreatic cancer (20 pts)	not analyzed	(1) CBR of 60% (2) reduction of CA19-9 serum level in 90% of pts (3) improvement in health-related quality of life

Fujiwara et al. [55] 2011	*nafamostat mesylate *	human pancreatic cancer cell lines (AsPC-1, BxPC-3, PANC-1)	down-regulation of IkB*α*, NF-*κ*B with reduction of ICAM-1, IL-8, VEGF, MMP-9, uPA	(1) increase of cell adhesion, programmed cell death (2) inhibition of angiogenesis, invasion, metastasis in peritoneal dissemination

Gocho et al. [56] 2013	(1)* nafamostat mesylate* (2)* nafamostat mesylate* *plus oxaliplatin *	human pancreatic cancer cell line (Panc-1) and pancreatic cancer mouse model	down-regulation of NF-*κ*B with reduction of ICAM-1, IL-8, VEGF, MMP-9, uPA, c-IAP1, c-IAP2	(1) increase of cell adhesion, caspase-8-mediated apoptosis (2) inhibition of PARP, angiogenesis, invasion and metastasis, (3) synergistic cytotoxic effect

PAR-2, Protease-activated receptor-2; MAPK, mitogen-activated protein kinase; COX, cyclooxygenase; IL, Interleukin; GM-CSF, Granulocyte-macrophage colony stimulating factor; TAT, Tumor-associated trypsinogen; IkB, Inhibitor of NF-*κ*B; NF-*κ*B, Nuclear factor-kappaB; MMPs, metalloproteinases; uPA, urokinase-type plasminogen activator; ICAM-1, Intercellular Adhesion Molecule-1, VEGF, Vascular endothelial growth factor, IAP, Inhibitors of apoptosis.

**Table 3:** All studies mentioned above that have considered tranilast.

Author, reference, year	Drug/s	Tumor target	Molecular mechanisms of action	Results
Chakrabarti et al. [57] 2009	*tranilast *	several mouse, rat and human breast cancer cell lines	(1) down-regulation of TGF-*β* pathway (2) inhibition of MAPK pathway	inhibition of tumor cell proliferation, angiogenesis, apoptosis, migration

Subramaniam et al. [58] 2010	*tranilast *	mouse breast cancer cell line (4T1)	(1) down-regulation of TGF-*β* pathway (2) induction cell arrest in the G_0_/G_1_ transition, PARP cleavage, AKT1 phosphorylation (3) up-regulation of p53 (4) reduction of ERK1/2 phosphorylation	inhibition of tumor cell proliferation, angiogenesis, apoptosis, migration

Hiroi et al. [59] 2002	*tranilast *	hamster pancreatic cancer cell line (PGHAM-1)	(1) down-regulation of TGF-*β* pathway with reduction of MMP-9 and VEGF levels (2) induction cell arrest in the G_0_/G_1_ transition	inhibition of tumor cell proliferation, angiogenesis

Mitsuno et al. [60] 2010	(1)* tranilast plus gemcitabine* (2)* gemcitabine *	human pancreatic cancer cell line (KP4)	decrease of RRM1 expression	(1) inhibition of tumor cell proliferation, angiogenesis, apoptosis (2) synergistic cytotoxic effect of combination therapy

Izumi et al. [61] 2009	*tranilast *	(1) prostate cancer cell lines and bone-derived stromal cells (2) SCID mice (3) advanced hormone-refractory prostaste cancer (21 pts)	down-regulation of TGF-*β*1 pathway	(1) induction of apoptosis (2) reduction of invasion and bone metastasis, PSA levels, improve prognosis

Noguchi et al. [62] 2003	*tranilast *	mouse model of oral squamous cell carcinoma	not analyzed	decrease of tumor growth, angiogenesis, cervical lymph node metastases

Yashiro et al. [63] 2003	*tranilast *	human gastric carcinoma cell line (OCUM-2D) and gastric fibroblast cell line (NF-10)	down-regulation of TGF-*β* pathway	decrease of tumor growth, angiogenesis, invasion

Platten et al. [64] 2001	*tranilast *	human malignant glioma cell line	down-regulation of TGF-*β*1-2 pathway	decrease of tumor growth, angiogenesis, migration, invasion

Nie et al. [65] 1997	*tranilast *	breast cancer cell lines (MCF-7)	induction cell arrest in the G_0_/G_1_ transition	decrease of tumor growth

Subramaniam et al. [66] 2011	*tranilast *	human breast cancer cell lines (triple positive-BT-474, triple negative-MDA-MB-231)	(1) up-regulation of p53 (2) induction cell arrest in the G_0_/G_1_ transition, AKT1 and ERK2 phosphorylation, PARP-cleavage product	induction of apoptosis, tumor growth, migration

Zhang et al. [67] 2009	*tranilast *	several ER negative human breast cancer cell lines	agonizing ARH with down-regulation of TGF-*β* and NF-*κ*B pathways	(1) induction of apoptosis (2) inhibition of angiogenesis, cell growth, invasion and metastasis

Hall et al. [68] 2010	*tranilast *	several human breast cancer cell lines	agonizing ARH with down-regulation of TGF-*β* and NF-*κ*B pathways	(1) induction of apoptosis (2) inhibition of angiogenesis, cell growth, invasion and metastasis

Isaji et al. [69] 1997	*tranilast *	human pancreatic cancer cell lines	decrease of VEGF and MMPs levels	inhibition of angiogenesis, cell growth, migration

TGF-*β*1, Tumor growth factor-beta1, MMPs, metalloproteinases; MAPK, mitogen-activated protein kinase uPA, PARP, poly ADP-ribose polymerase; urokinase-type plasminogen activator; AKT1, RAC-alpha serine/threonine-protein kinase; ERK, Extracellular regulated kinase 2; VEGF, Vascular endothelial growth factor; RRM1, Ribonucleotide reductase M1.
